# Choanal atresia repair in Europe and the world – a comprehensive investigation of the current state of care

**DOI:** 10.1007/s00405-026-10319-4

**Published:** 2026-06-01

**Authors:** Miray-Su Yılmaz Topçuoğlu, Patrick J. Schuler, Claire Hopkins, Maryana Cherkes, Marek Ciller, Sara Costa, Natalia Glibbery, Petra Kovács, Luiza Mitrea-Sireţeanu, Jakub Zieliński, Jens H. Westhoff, Ingo Baumann

**Affiliations:** 1 Department of Otorhinolaryngology, Head and Neck Surgery, Medical Faculty Heidelberg, University Hospital Heidelberg, Im Neuenheimer Feld 400, Heidelberg, 69120 Germany; 2 Ear, Nose and Throat Department, Guy’s Hospital, London, UK; 3 Department of Otorhinolaryngology, Head and Neck Surgery, Medical University of Graz, Graz, Austria; 4 Department of Otorhinolaryngology, Second Faculty of Medicine, University Hospital Motol, Prague, Czech Republic; 5 Department of Otolaryngology-Head and Neck Surgery, Unidade Local de Saúde de Santo António, Porto, Portugal; 6 Department of Otolaryngology, Addenbrooke’s Hospital, Cambridge University Hospitals NHS Foundation Trust, Cambridge, UK; 7 Department of Otolaryngology, Heim Pál National Pediatric Institute, Budapest, Hungary; 8 Saint Mary Plaza Clinic, Bucharest, Romania; 9 Department of Otolaryngology, Head and Neck Surgery, Wroclaw Medical University, Wroclaw, Poland; 10 Department I, Center for Pediatric and Adolescent Medicine, Medical Faculty Heidelberg, University Hospital Heidelberg, Heidelberg, Germany

**Keywords:** Choanal atresia, Congenital abnormalities, Diagnosis, Postoperative care, Survey

## Abstract

**Purpose:**

Management of patients with choanal atresia (CA) involves various aspects. While a recent study revealed variability in CA management practices across German Ear, Nose, Throat (ENT) departments, the actual current state of care in Europe and worldwide remains to be elucidated, which was the aim of this study.

**Methods:**

An online survey was conducted on preoperative diagnostics, surgical procedures, and postoperative care in CA patients. A total of 116 ENT departments from 46 countries and six continents participated.

**Results:**

The median number of CA repairs performed annually across all continents was three. Unilateral CA repair was performed by 64% of ENT departments in patients under five years and by 30% in patients over five years. Preoperative hearing tests were not standard (19%). Computed tomography (CT) was often performed (84%). Endoscopic transnasal CA repair was the main approach (81%). The posterior vomer was routinely resected by 57% of participants. Stents were always or mostly used by 33%. Saline irrigations (77%), topical corticosteroids (37%), and perioperative antibiotics (32%) were recommended as postoperative nasal care measures. Recurrences were attributed to scarring (71%), granulation tissue (40%), and insufficient vomer resections (21%).

**Conclusion:**

While some practices align with existing recommendations, other aspects require further discussion. The age at which patients should undergo unilateral CA repair still needs clarification. Preoperative hearing tests were not commonly implemented. CT imaging was widely used despite the associated radiation exposure. Over half of the participants resected the posterior vomer. The use of stents and perioperative antibiotics should be discussed further.

## Introduction

The management of patients with congenital choanal atresia (CA) is a challenging and uncommon condition for Ear, Nose, Throat (ENT) surgeons [1]. On average, three surgical CA repairs are performed in German ENT departments each year [2], reflecting the limited experience individual surgeons might have with this condition. Clearly defined workflows are useful for optimising care in such rare cases [1, 3, 4].

A high-quality consensus paper and a Slovenian national recommendation list for managing CA patients have been published [4, 5]. However, actual practice often deviates from these recommendations. A previous preliminary German study from 2024 only focused on stents and posterior vomer resections [6], whereas another recently published study investigated a broad range of aspects of CA management, from preoperative diagnostics to postoperative care, in order to assess the current standard of CA patient care in Germany [2]. Overall, there was clear potential for improvement in the management of CA in Germany, particularly with regard to preoperative imaging, assessment of hearing status, surgical techniques, and postoperative nasal care [2]. However, the single-nation design limited these findings to German ENT departments. As the optimal management of CA patients should be consistent across different countries, it is important to follow up on the previous work by investigating the current state of care available to these patients, not only in Germany, but worldwide.

The consensus paper by Moreddu et al. was based on recommendations from members of the International Pediatric Otolaryngology Group across eight different countries [4]. Additionally, Urbančič et al. published national recommendations for the management of CA, based on the recommendations of a national expert council in Slovenia [5]. To date, no further investigations have been published examining real-world CA management practices from an international perspective. This study aimed to address this gap by providing a comprehensive international overview of current CA management practices, encompassing preoperative diagnostics, surgical techniques, and postoperative care. This will facilitate valuable comparisons, identify areas for improvement and foster further discussion within the international paediatric ENT community.

## Materials and methods

An online survey, available in English and German, was conducted. The European Rhinologic Society (ERS) and the European Society of Pediatric Otorhinolaryngology (ESPO) distributed the survey link to their subscribed members via society newsletters. Simultaneously, the authors also emailed the link to ENT surgeons within their professional networks.

The study period was from July 2025 to November 2025. The online survey tool LimeSurvey (version 6.13.2 + 250506, LimeSurvey GmbH) was employed. Similar to a former study, different demographic, preoperative, surgical, and postoperative aspects with regard to the ENT-related management of patients with congenital CA were queried [2]. The queried survey items are listed in Table 1. The results of the survey were contrasted to previous recommendations from the international consensus paper by Moreddu et al. [4] and Urbančič et al. [5] (Table 1). The study did not involve single patient data.

**Table 1:** Comparison of recommendations from an international consensus paper and a national guideline to this study’s real-world data

	International consensus paper Moreddu et al. [4]	National guideline Urbančič et al. [5]	Real-world data
Participants/Design	Consensus paper; 28 IPOG-members in 22 tertiary-care centre departments from 8 countries	National guideline; Authors of Slovenian national guideline	Survey study; 116 participants from 46 countries
Number of CA repairs/year	N/A	N/A	3.0; 0–50; 7
Size of ENT departments	N/A	N/A	0–20 beds (41%) to > 100 beds (16%)
Age recommendation for elective repair of unilateral CA	− After the age of at least 6 months (92.8% agreement) − One year (78.5% agreement) − Two years (36% agreement) − High symptom burden may lead to earlier surgery (no percentage of agreement given)	− Recommended after the infant is six months old	− 0–6 months: 19%; 7–12 months: 7%; 1–2 years: 12%; 2–5 years: 26%; 5–8 years: 10% − 8–11 years: 7%; further details in Table 2
Preoperative diagnostics			
Rhinologic assessment	− Nasofiberscopy/Endoscopy in all patients (85.7% agreement) − Insertion of nasogastric tube in selected cases (53.6%) − Mirror or paper examination under the nostril in all patients (39.3% agreement)	− Strongly recommended otorhinolaryngological examination with examination of nose and face, anterior rhinoscopy, toilet, anemization, epimucosal anaesthesia of the nose and examination with a flexible or rigid endoscope	− Flexible nasal endoscopy performed by 81% of participants
Audiologic assessment	− Audiologic assessment should be performed in all patients with CA (57.1% agreement), using adapted method	− An audiovestibulological evaluation is strongly recommended for hearing assessment	− Tympanometry performed by 19% and otoacoustic emissions additionally or instead of tympanometry performed by 17% of participants
Radiologic assessment	− Each patient should undergo preoperative CT scan (100% agreement)	− CT and head ultrasound strongly recommended for bilateral CA; recommended for unilateral CA	− 84% of participants performed preoperative CT scans, 16% performed MRI scans
Genetic testing	− Genetic consultation in all patients (35.7% agreement) − Selective request for genetics consultation (64.3% agreement)	− Strongly recommended to exclude congenital abnormalities in bilateral CA by involvement of a clinical geneticist	− Up to 22% of participants performed preoperative genetic tests
Surgical approach	− Endoscopic approach for all patients (89.3% agreement) or only in selected cases (10.7% agreement) − Transpalatinal approach only in selected cases (92.9% agreement)	− Transnasal endoscopic approach strongly recommended for unilateral and bilateral CA − Transpalatinal resection not recommended	− 81% of participants performed transnasal endoscopic CA repair
Surgical technique	− Never elevate mucosal flaps (64.3% agreement), no statement on vomer resection	− N/A	− 57% resected posterior vomer, 48% elevated mucosal flaps
Add-ons			
Stents & removal	− Stenting in selected cases for bilateral (53.6% agreement) and unilateral (60.7% agreement) CA − Stenting all patients in bilateral (32.1% agreement) and unilateral (14.3% agreement)	− Recommendation: if used, stents should be removed within 7 days − The extent of resection in unilateral CA is strongly recommended to be large enough so that no stent is needed	− 40% never/hardly used stents, 33% always/mostly used stents − Stent removal median 10 days after surgery
Packing	− N/A	− N/A	− 21% performed soft packing if necessary
Mitomycin C	− Never used (82.1% agreement) − Used for refractory CA (42.9% agreement), no use for recurrences (32.1% agreement)	− N/A	− 7% used Mitomycin C, (half of them use it only for revision cases)
Drug-eluting stents	− N/A	− N/A	− 5% used drug-eluting stents, not common yet
Length of postoperative stay after elective unilateral CA repair	− N/A	− Discharge on first postoperative day recommended	− 2–3 nights (35%) or one night (30%) of postoperative surveillance
Postoperative nasal care measures			
Saline irrigations	− Saline application (100% agreement) for up to 4 weeks (82.1% agreement), nasal corticosteroids used (71.4% agreement)	− Strongly recommended daily administration of saline solution drops and nasal corticosteroids for two months	− 77% recommended saline irrigations
Topical corticosteroids			− 37% recommended topical corticosteroids
Perioperative antibiotics	− N/A, no postoperative antibiotics (53.6% agreement)	− N/A, antibiotic nose drops recommended in purulent discharge	− 32% recommended perioperative antibiotics
Daily suctioning	− N/A	− N/A	− 24% recommended daily suctioning of nose
Complications	− N/A	− N/A	− 64% granulation tissue, 24% epistaxis
Recurrences attributed to	− N/A	− N/A	− Scarring (71%), granulations tissue (40%), insufficient vomer resection (21%)

The table compares the aspects of the present study, listed here in the “Real-World Data” column, to the recommendations provided in the IPOG (International Pediatric Otolaryngology Group) consensus paper by Moreddu et al. [4] and the published national guideline recommendations by Urbančič et al. [5] to make it possible to contrast the real-world data with existing recommendations/guidelines. In the left, first column all queried aspect of the underlying study are listed. Number of CA (choanal atresia) repairs/year given as median; range; interquartile range. *N/A* no corresponding statements/recommendations available. *CT* computed tomography, *MRI* magnetic resonance imaging. Perioperative antibiotics refer to systematically administered antibiotics at surgical tie and two days afterwards.

Allowing for anonymous participation was intended to control for interviewer and response biases. Data analysis and visualisation were performed using GraphPad Prism (version 10.4.0, GraphPad Software, Boston, Massachusetts, USA). Continuous data were presented as the median, minimum-to-maximum range, and interquartile range (IQR). To reduce bias arising from the differing number of participants per continent, the results were reported as percentages where appropriate, showing proportions rather than absolute numbers. The Kruskal–Wallis test was used to analyse differences between continents, followed by Dunn’s post hoc test with adjustment for multiple comparisons. The significance level was set at *p* < 0.05.

## Results

### Participation and geographic distribution

A total of 116 ENT departments from 46 different countries around the world voluntarily participated in the online survey (Table 2). Apart from two participants from Egypt and two from the United Kingdom who each worked in the same ENT department, all the other 112 participants came from different ENT departments. All responses were included. The number of responses to each question ranged from a minimum of 91 to a maximum of 116. The 46 countries were divided into six subgroups based on their geographical location: Africa, Asia, Europe, North America, Oceania, and South America (Table 2). The size of ENT departments ranged from 0 to 20 beds (41%, 47/116) to over 100 beds (16%, 19/116).

**Table 2:** Participating countries and perioperative treatment aspects

Group	Total	Africa	Asia	Europe	North America	Oceania	South America
Countries: n (%)	46 (100)	5 (11)	10 (22)	23 (50)	3 (7)	2 (4)	3 (7)
ENT departments: n (%)	116 (100)	11 (9)	18 (16)	71 (61)	5 (4)	5 (4)	6 (5)
Name of participating country (total number of participants of each country)		Algeria (1), Egypt (5), Libya (2), Nigeria (1), Uganda (2)	India (4), Iraq (1), Israel (1), Malaysia (1), Oman (1), Pakistan (2), Qatar (1), Saudi Arabia (1), Turkey (5), Vietnam (1)	Austria (3), Belgium (1), Bosnia and Herzegovina (1), Bulgaria (2), Czech Republic (2), Denmark (2), Finland (2), France (1), Germany (5), Greece (6), Hungary (1), Ireland (2), Italy (5), Netherlands (2), Norway (4), Poland (2), Portugal (5), Romania (1), Spain (4), Sweden (3), Switzerland (2), Ukraine (1), United Kingdom (14)	Canada (1), Mexico (1), United States (3)	Australia (4), New Zealand (1)	Brazil (3), Chile (2), Colombia (1)
Numbers of CA repairs/year	*n* = 115 3.0 (0–50; 7)	*n* = 11 15.0 (2–48; 22)	*n* = 18 3.5 (0–50; 4)	*n* = 70 2.0 (0–25; 4)	*n* = 5 3.0 (0–8; 5)	*n* = 5 4.0 (2–6; 3)	*n* = 6 2.0 (2–15; 13)
Age recommendation for elective repair of unilateral CA: n (%) 0–6 months 7–12 months 1–2 years 2–5 years 5–8 years 8–11 years 11–14 years 14–17 years 17–20 years >20 years Other	*n* = 101 19 (19) 7 (7) 12 (12) 26 (26) 10 (10) 7 (7) 4 (4) 2 (2) 2 (2) 5 (5) 7 (7)	*n* = 11 2 (18) 0 (0) 1 (9) 2 (18) 4 (36) 1 (9) 0 (0) 0 (0) 0 (0) 0 (0) 1 (9)	*n* = 17 4 (24) 2 (12) 1 (6) 3 (18) 3 (18) 2 (12) 0 (0) 0 (0) 0 (0) 2 (12) 0 (0)	*n* = 58 9 (16) 4 (7) 9 (16) 14 (24) 3 (5) 4 (7) 4 (7) 2 (3) 2 (3) 2 (3) 5 (9)	*n* = 4 0 (0) 1 (25) 1 (25) 1 (25) 0 (0) 0 (0) 0 (0) 0 (0) 0 (0) 1 (25) 0 (0)	*n* = 5 1 (20) 0 (0) 0 (0) 4 (80) 0 (0) 0 (0) 0 (0) 0 (0) 0 (0) 0 (0) 0 (0)	*n* = 6 3 (50) 0 (0) 0 (0) 2 (33) 0 (0) 0 (0) 0 (0) 0 (0) 0 (0) 0 (0) 1 (17)
Time from surgery to stent removal [days]	*n* = 24 10.0 (2–42; 8)	*n* = 3 21.0 (6–42; 36)	*n* = 3 14.0 (7–30; 23)	*n* = 10 12.0 (7–42; 10)	*n* = 3 7.0 (7–14; 7)	*n* = 4 8.5 (7–14; 6)	*n* = 1 2.0 (2–2; 0)
Utilisation of P, MC, DES: n (%)	*n* = 106 P: 22 (21) MC: 7 (7) DES: 5 (5)	*n* = 11 P: 1 (9) MC: 1 (9) DES: 0 (0)	*n* = 17 P: 5 (29) MC: 4 (24) DES: 1 (6)	*n* = 62 P: 15 (24) MC: 1 (2) DES: 4 (6)	*n* = 5 P: 1 (20) MC: 0 (0) DES: 0 (0)	*n* = 5 P: 0 (0) MC: 1 (20) DES: 0 (0)	*n* = 6 P: 0 (0) MC: 0 (0) DES: 0 (0)

Absolute numbers n and in brackets relative numbers in percent [%] are given for the participating countries, the ENT departments, age recommendations for elective repair of unilateral CA, and utilisation of P, MC, DES. Names of the participating countries are presented with the number of participants per country in brackets. Numbers of CA repairs/year and time from surgery to stent removal are given as median (minimum to maximum range; interquartile range). For numbers of CA repairs/year, age for elective repair of unilateral CA, time from surgery to stent removal, and utilisation of P, MC, DES, in the first row the total number of responses are presented. *ENT* Ear-Nose-Throat, *CA* choanal atresia, *P* packing, *MC*: Mitomycin C, *DES* drug-eluting stent

### Choanal atresia repairs are rare and there is no uniform age recommendation for elective repair of unilateral choanal atresia

**Fig. 1:**
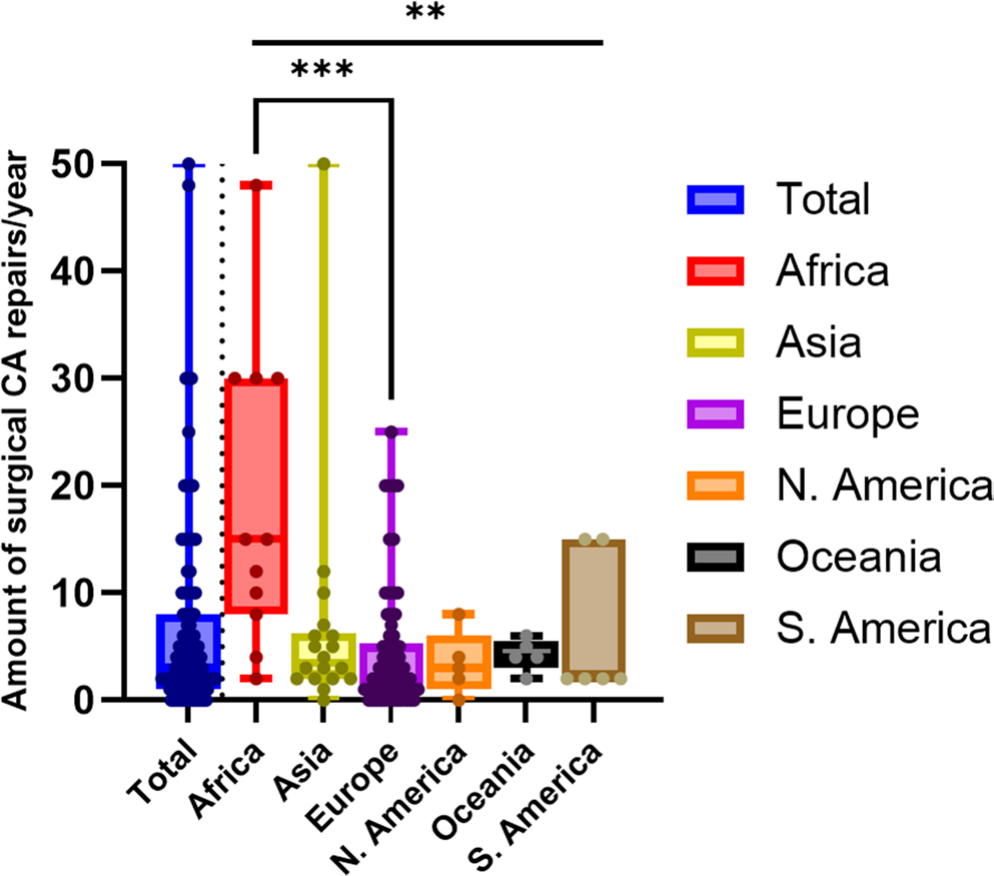
Annual number of performed choanal atresia repairs. The absolute number of performed surgical choanal atresia (CA) repairs per year is shown for all participating countries, in total (total number of responses given: *n* = 115) and subgrouped by continent (number of responses given: Africa: *n* = 11; Asia: *n* = 18; Europe: *n* = 70; North America: *n* = 5; Oceania: *n* = 5; South America: *n* = 6). N.: North, S.: South. ** *p* < 0.01; *** *p* < 0.001

In total, a median of 3.0 CA repairs per year were performed (range: 0–50; IQR: 7; 115 responses). The annual number of CA repairs performed differed significantly between all continents in the Kruskal-Wallis test (*p* < 0.01). Particularly, African ENT departments reported a significantly higher annual number of CA repairs than European ENT departments, as measured by Dunn’s post hoc test (*p* < 0.001, Fig. 1; Table 2). There was a significant and broad variation among all participating ENT departments in the recommended age for elective repair of unilateral CA (*p* < 0.001, Table 2). Of the participating ENT departments from all over the world, 64% recommended an age below five years for elective unilateral CA repair, and of these, 26% recommended an age of younger than 12 months. About 30% recommended an age of over five years (Table 2).

### Preoperative flexible nasal endoscopy and computed tomography are common, whereas hearing assessment is uncommon

**Fig. 2:**
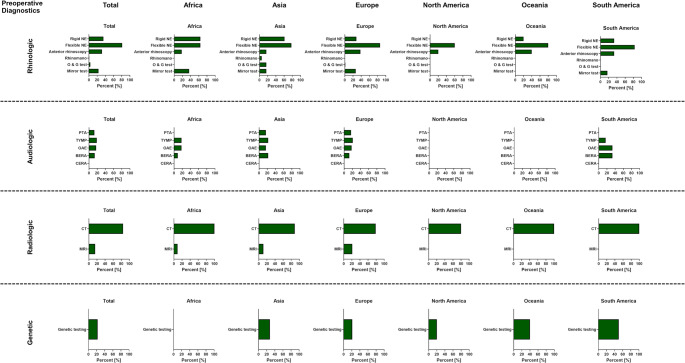
Preoperative diagnostic assessments in patients with choanal atresia. The percentages of rhinologic, audiologic, radiologic and genetic diagnostic modalities are presented for all participating countries, in total (*n* = 116) and subgrouped by continent (Africa: *n* = 11; Asia: *n* = 18; Europe: *n* = 71; North America: *n* = 5; Oceania: *n* = 5; South America: *n* = 6). No audiological assessments were reported by participants from North America and Oceania, and no genetic testing was reported by African participants. NE: nasal endoscopy; Rhinomano: rhinomanometry; O & G test: Olfactory and gustatory test; PTA: Pure Tone Audiometry; TYMP: Tympanometry; OAE: Otoacoustic Emissions; BERA: Brainstem Evoked Response Audiometry; CERA: Cortical Evoked Response Audiometry; CT: Computed Tomography; MRI: Magnetic Resonance Imaging

The most common rhinological clinical examination was flexible nasal endoscopy across all continents (81%, 94/116; Fig. 2). Preoperative hearing tests were not widely established across all continents, with a maximum of 19% (22/116) performing tympanometry (Fig. 2). The majority of participating ENT departments performed preoperative CT scans (84%, 98/116), while 16% (18/116) performed preoperative magnetic resonance imaging (MRI) (Fig. 2). Up to 22% (26/116) of the participating ENT departments include genetic tests as part of their preoperative diagnostic measures (Fig. 2). No significant differences were observed in the distribution of preoperative diagnostics performed (rhinologic, audiologic, radiologic, genetic) across different continents.

### Transnasal endoscopic choanal atresia repair was the approach of choice, and resection of the posterior vomer and elevating mucosal flaps were not routine

**Fig. 3:**
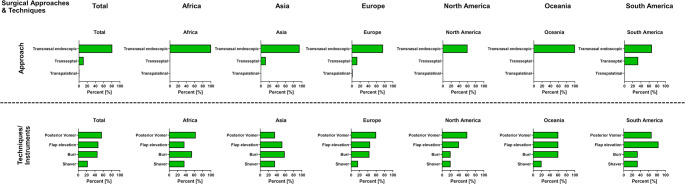
Surgical approaches and techniques used for choanal atresia repair. The upper row illustrates surgical approaches, while the lower row refers to applied techniques and instruments. Data are shown for the participating countries, in total (total number of responses given: *n* = 106) and subgrouped by continent (number of responses given: Africa: *n* = 11; Asia: *n* = 17; Europe: *n* = 62; North America: *n* = 5; Oceania: *n* = 5; South America: *n* = 6). ‘Posterior vomer’ refers to the resection of the posterior vomer. ‘Flap elevation’ refers to the elevation of mucosal flaps in order to cover exposed bone

Overall, 81% (86/106) of ENT departments across all continents primarily used the transnasal endoscopic approach for CA repair, followed by the transseptal approach (11%, 12/106; Fig. 3). Only one ENT department (1%, 1/106) performed the transpalatinal approach.

Approximately half of the participating ENT departments regularly employed resection of the posterior vomer (57%, 60/106) and elevation of a mucosal flap (48%, 51/106; Fig. 3). There was no difference across the continents. The burr (46%, 49/106) and, to a lesser extent, the shaver (23%, 24/106) were used as supporting instruments by the ENT departments in all countries (Fig. 3). No significant technique- or instrument-related difference was found between the continents. Several ENT departments independently reported on their use of otosurgical instruments and backbite forceps in the free-text answer option. Three ENT departments in the UK and one in Australia reported performing a combined transoral and transnasal approach for neonates with bilateral CA.

### Stent use is prevalent all over the world, further materials are rarely used

**Fig. 4:**
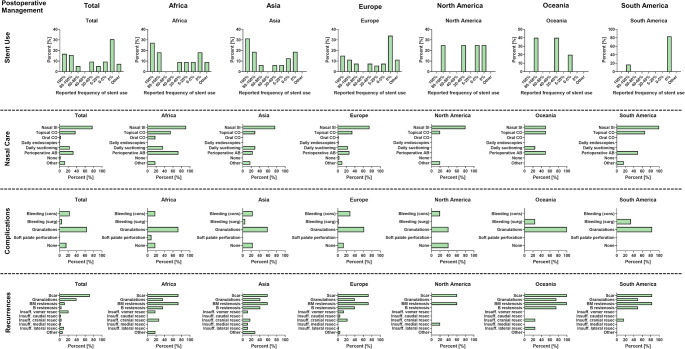
Postoperative management in patients with choanal atresia. The reported frequency of stent use (total number of responses given: *n* = 95; Africa: *n* = 11; Asia: *n* = 16; Europe: *n* = 53; North America: *n* = 4; Oceania: *n* = 5; South America: *n* = 6), the recommended postoperative nasal care measures (total number of responses given: *n* = 102; Africa: *n* = 11; Asia: *n* = 17; Europe: *n* = 59; North America: *n* = 5; Oceania: *n* = 4; South America: *n* = 6), the observed postoperative complications and aspects attributed to causing recurrences (total number of responses given: *n* = 101; Africa: *n* = 11; Asia: *n* = 17; Europe: *n* = 58; North America: *n* = 5; Oceania: *n* = 4; South America: *n* = 6) are shown for all participating countries, in total and subgrouped by continent. The frequencies of stent use were ordered as follows: 100%: always; 80–100%: mostly; 60–80%: frequently; 40–60%: often; 20–40%: sometimes; 5–20%: occasionally; 0–5%: hardly; 0%: never; other. SI: saline irrigations; CO: corticosteroids; AB: antibiotics; cons: conservative management possible; surg: surgical management needed; Granulations: granulation tissue growth; BM: bony-membranous; B: bony; Insuff.: insufficient; resec: resection

In total, 40% never (29/95) or hardly (9/95) used stents, and 33% always (16/95) or mostly (15/95) used stents across all continents (Fig. 4). While in Europe (14/53), North (1/4) and South (1/6) America 17–26% of the participating ENT departments always or mostly used stents, this was more common for the African (5/11), Asian (8/16), and Oceanian (2/5) ENT departments with 40–50% (Fig. 4). If stents were used, they were reported to be removed after a median of 10 days after surgery across all continents (range 2–42; IQR: 8; Table 2). In the free-text answer option, four participants described the use of stents for bilateral and revision cases, but not for unilateral cases. The stent materials reported were silicone sheets, splints, endotracheal tubes, and nasogastric tubes. Topical mitomycin C application was performed by 7% (7/106), postoperative soft packing was employed by 21% (22/106) if necessary, and drug-eluting stents was used by 5% (5/106) across all continents (Table 2). In the free-text answer option, it was reported that the use of mitomycin C was reserved for revision cases only, where it was used as a topical agent. Drug-eluting stents were described as having only been used in extensive revision cases, and one ENT department reported having just started using drug-eluting stents postoperatively, with little experience yet.

### Length of postoperative stay after elective unilateral CA repair and recommended postoperative nasal care measures

The most common length of postoperative stay for elective repair of unilateral CA in children was two to three nights as reported by 35% (32/91) of the participating ENT departments. One night of postoperative surveillance (30%, 27/91) or day surgery (13%, 12/91) was also reported, depending on prevalent comorbidities.

The most frequently recommended postoperative care measures were nasal saline irrigations (77%, 79/102), followed by topical corticosteroids (37%, 38/102), perioperative antibiotics (32%, 33/102) and daily suctioning of the nose (24%, 24/102; Fig. 4).

The recommended duration of postoperative nasal care was 5–8 weeks (39%, 36/92) or 2–5 weeks (25%, 23/92) across all participating ENT departments. Across all continents, responsibility for postoperative follow-up care rested with the ENT department that performed the surgical CA repair (71%, 72/102), the attending ENT specialist at the outpatient facility (25%, 26/102), and to a lesser extent, the attending paediatrician (2%, 2/102).

### Recurrences were attributed to scarring, granulation tissue, and insufficient vomer resections

Across all continents, the most frequent postoperative complications were granulation tissue growth (64%, 65/101) and conservatively manageable, self-limiting postoperative bleeding (24%, 24/101), with the same distribution within the different subgroups (Fig. 4).

Across all continents, recurrences were attributed to scarring (71%, 72/101), granulation tissue (40%, 40/101) and insufficient posterior vomer resection (21%, 21/101; Fig. 4).

## Discussion

This study examined the current clinical practices for managing CA in ENT departments across Europe and the world, in light of existing data from Germany [2] and recommendations from previous consensus/guideline works [4, 5]. The aim was to enable comparisons, raise awareness of areas in need of improvement, and facilitate further discussion on this important topic.

Encouragingly, 116 ENT departments from 46 countries and six continents participated in the survey. Nevertheless, the results from the presented study cannot be globalised, as not all countries participated and the distribution of the participating ENT departments did not mirror the global demographic distribution. For instance, while a prevailing 71 ENT departments from Europe participated in the study, only six did so from South America, and just five from North America and Oceania. The European predominance can be attributed to the distribution method of the survey link. In future, the scope could be expanded by including more paediatric ENT and rhinology societies from all around the world. Furthermore, the survey being in English or German limited the pool of participants to English or German speakers, which unfortunately limited the inclusiveness of this study.

ENT departments of all sizes reported managing patients with CA, demonstrating that even smaller centres encounter these rare cases. This highlights the need for thorough training for all ENT surgeons. Centralising CA repairs in specialist centres could increase the number of cases, allowing for more consistent and routine care of this vulnerable patient group.

First, the number of CA repairs performed and the age recommendations for elective repair of unilateral CA were investigated. In line with the previous study in Germany [2], a median of three CA repairs/year indicated that CA repair is scarce. African ENT departments reported the highest median number of CA repairs per year. Although this may suggest a higher number of affected patients in the African continent [7], the observed differences are more likely to be due to variations in healthcare system structures, referral patterns, and centre specialisation, rather than to genuine differences in disease prevalence. However, as only eleven African ENT departments participated, most of which were located in North Africa, the data do not allow for a representative assessment of the whole African continent.

As in the previous study on CA management in Germany [2], age recommendations for elective repair of unilateral CA were heterogeneous. Although there was a trend towards younger patients under the age of 5 years, it remains unclear how young patients should be to undergo elective unilateral CA repair in this study. Unfortunately, there is still a lack of high-quality evidence on the isolated impact of delayed CA repair on aspects of otological, sinonasal, and olfactory health, craniofacial growth and quality of life. However, early CA repair is recommended to prevent the atresia plate from thickening [8], reduce chronic sinonasal and otologic infections [9], preserve olfactory function [10, 11], support craniofacial growth [12, 13], and improve quality of life and function [14]. Conversely, delaying surgery allows for better anatomical access and increased anaesthetic safety [15]. Based on these pragmatic considerations, and despite the lack of evidence-based data, the consensus paper by Moreddu et al. (92.8% agreement) and the Slovenian national recommendations presented by Urbančič et al. recommend the elective repair of unilateral CA after the age of at least six months, ideally performing the procedure until the age of 12 months (78.5% agreement in [4], Table 1) [4, 5]. This is in contrast to the observations made by this study, in which most participants stated that they performed elective CA repair in 2–5-year-old patients. So conclusively, following the existing recommendations [4, 5], the age range should be decreased to 6- to 12-month-old patients. However, for patients with unilateral CA, the symptom burden remains a key factor in determining the timing of CA repair, as unilateral CA repair is not an emergency procedure, in contrast to CA repair for bilateral cases [1, 4].

Despite the lack of an evidence base, in line with current practical considerations and recommendations (Table 1) [4, 5], preoperative diagnostics mainly relied on flexible nasal endoscopy, while rigid nasal endoscopy, rhinomanometry, olfactory and gustatory testing, and the mirror test played only a minor role. Flexible endoscopy provides good visualisation [16, 17] and is minimally invasive and cost-effective. Passing a nasal catheter is common practice in newborns with bilateral CA, but this method does not allow visualisation. Compliance can be challenging, requiring careful guidance of both children and their parents.

Although preoperative hearing tests adapted to the patient’s age and function were strongly recommended by the national guideline from Urbančič et al. [5], and 57% of the participating members in the consensus paper from Moreddu et al. also agreed on preoperative audiologic assessments (Table 1) [4], only 19% of the participants conducted any audiological assessment, which is lower than the 41% reported in the previous German study [2]. While newborn hearing screening can detect early impairments [18, 19], ongoing testing throughout childhood can identify later hearing loss and allow for timely intervention. For instance, conductive hearing loss resulting from tympanic effusions can be treated with paracentesis, ventilation tubes and/or adenoidectomy during elective CA repair. Preoperative age- and development-adapted hearing tests should therefore become routine in CA management to ensure comprehensive care.

Compared to recently published data from Germany, where 52% of ENT departments performed preoperative CT scans [2], 84% of departments worldwide routinely performed preoperative CT scans. CT scans support the diagnosis and surgical planning [1, 3–5] and enhance surgical safety [20, 21]. In the eyes of the authors, they are particularly valuable and indispensable in syndromic, bilateral or revision cases. Furthermore, CT scans should be considered in cases where complex anatomy and difficult surgical conditions are expected. However, arguments against routine preoperative CT scans include the associated radiation exposure [22], the fact that sufficient visualisation can be achieved through nasal endoscopy [16, 17, 21], and the potential need for anaesthesia in young children to obtain sufficient CT images. Taking these considerations into account, the authors propose that CT scans can be avoided in patients with non-syndromic, unilateral CA and normal craniofacial anatomy. These statements partly oppose, and partly align with existing recommendations. While the consensus paper by Moreddu et al. recommended preoperative CT scans without exception [4], the Slovenian national recommendations presented by Urbančič et al. strongly recommended them for bilateral cases but made a weaker recommendation for unilaterally affected patients (Table 1) [5]. Although less common, MRI scans can provide complementary anatomical information. Preoperative imaging should therefore be considered on a case-by-case basis, weighing up the diagnostic benefits against the risks and the patient’s individual circumstances.

Preoperative genetic testing was performed by only 3% of German ENT departments [2] and none of the African departments, compared to up to 22% in other continents. From the authors’ perspective, to save resources, genetic testing should generally focus on bilateral CA or cases with suspected syndromic comorbidity, as it has been recommended earlier (Table 1) [4, 5]. Of note, in some countries, testing may also be arranged by paediatricians before an ENT referral and is not under the responsibility of the ENT departments.

Next, the study focused on the surgical techniques applied for CA repair from the participating ENT departments. As recommended [3–6, 9], transnasal endoscopic CA repair was the preferred approach in the majority of the participating ENT departments as a safe procedure with good visualisation and a low risk profile [9, 23–26]. Interestingly, although insufficient vomer resections have been attributed to cause recurrence [9, 27–29], and 21% of the ENT departments participating in the current study reported this, only 57% performed posterior vomer resections. However, evidence on the role of posterior vomer part resections for recurrences is low [9, 29], and no official recommendations exist on this topic (Table 1). The authors want to increase international awareness on the importance of sufficient posterior vomer resections to potentially decrease recurrence rates.

Although mucosal flap elevation is not yet widespread [4], it is used almost twice as often internationally (48%) as in Germany (25%) [2], reflecting an internationally growing trend towards using mucosal flaps for CA repair [9, 17, 25–28, 30–39]. Mucosal flaps may improve healing, reduce granulation and restenosis [9, 17, 25–28, 30–39], but their elevation can be very challenging [1]. Further studies on long-term outcomes are therefore warranted, as the existing evidence base is currently very limited. Moreddu et al. did not recommend flaps for the majority (64.3%, Table 1) [4], and Urbančič et al.‘s recommendation list did not include a specific statement on mucosal flaps [5].

The use of stents following CA repair, as well as the duration of their use, varied depending on the continent. Overall, the number of ENT departments with hardly any to no stent use (40%) was higher than the number of departments that used stents always or mostly (33%). However, stent use was more common in African and Asian ENT departments. As stents can cause complications, enhance granulation tissue growth and increase the risk of restenosis as several evidence-based meta-analyses have shown [40–42], their use should be restricted to exceptional cases only according to various investigations [4–6, 16, 43]. Also, Moreddu et al. recommend that stents should only be used for selected cases [4].

For elective, uncomplicated cases, postoperative in-hospital stay was brief or performed as day surgery, as recommended by Urbančič et al. [5]. Given the low risk profile of CA repairs [9, 24, 28, 44], expanding day surgery based on patient risk profiles could ease family management and reduce hospital exposure for children. The participating ENT departments reported that postoperative complications were rare, tending to involve granulation tissue or minor, self-limiting bleeding, as was also reported by German ENT departments [2].

Despite the lack of evidence-based data, but in line with former recommendations (Table 1) [4, 5, 9], postoperative saline irrigations, and topical corticosteroids for up to eight weeks were administered by most of the participating ENT departments. However, a total of 32% of all participating ENT departments and up to 73% of African ENT departments also administered perioperative antibiotics for CA repair. There is no evidence-based, high-quality data on the perioperative use of antibiotics in CA repair. However, the results of this study clearly contradict existing recommendations. Urbančič et al. only recommended nasal antibiotic drops in cases of purulent discharge following surgery, and did not comment on the use of systemic antibiotics (Table 1) [5]. In their consensus paper, Moreddu et al. presented that there was no clear recommendation for or against the postoperative systemic use of antibiotics in CA repair (Table 1) [4]. As with sinus surgery, perioperative antibiotics could be reduced for uncomplicated CA repairs [45].

The study had several limitations. In addition to the aforementioned continent-related selection bias and lack of global generalisability, given that the data were primarily from Europe, a further major limitation was that it included both unilateral and bilateral CA, despite the fact that they differ in terms of airway and surgical time management and comorbidities. Nevertheless, both CA types were included in a single survey in order to avoid overburdening participants with two separate requests and to ensure sufficient participation rates. However, since ENT surgeons encounter both unilateral and bilateral CA in real practice, this study offers valuable insight into the general CA management, enabling readers to reflect on their own practices. As part of the data analysis, the different continents were compared with each other. However, as participation rates varied considerably, with fewer participants from North America, Oceania, and South America, these comparisons should be interpreted with caution. Furthermore, it should be noted that individual surgeons’ responses may not fully reflect the practices of their departments or country and that current practice does not necessarily align with evidence-based recommendations. In this context, it should be noted that due to the rarity of choanal atresia, there is a limited amount of high-quality, evidence-based research on the condition’s management. This makes it necessary to exercise caution when comparing existing data. Future studies should aim for broader and more balanced international representation of ENT departments, in order to distinguish general trends more clearly from individual preferences and to overcome the continent-related selection bias of this study, which had a predominant European participation.

## Conclusion

This is the first international study that assesses the real-world care of CA patients, providing valuable insights for all clinicians involved. While some practices align with existing recommendations, other aspects require further discussion. Firstly, although there was no clear trend regarding the optimal age for elective unilateral CA repair, the age range at which the elective surgery is performed should generally be reduced further, following existing recommendations, to between 6 and 12 months of age. Secondly, preoperative hearing tests were not widely performed, even though they are recommended. The authors advise increasing the number of routinely performed preoperative age- and development-adapted hearing tests to raise the identification rate of those in need of audiological intervention. Thirdly, although preoperative CT scans were common, as recommended by various authors, their necessity should be critically weighed against the associated radiation exposure, and their use should depend on the underlying disease constellation (e.g., bilateral, syndromic, revision, expected complex anatomy) with MRI scans being a potential alternative. Fourthly, the role of mucosal flaps is still unclear and requires further investigation. Fifthly, since previous investigations and the observations of this study’s participants suggest that recurrences may be attributed to inadequate posterior vomer resections, the authors advise increasing the use of posterior vomer resections. Sixthly, around one-third of departments routinely used stents. This practice should be limited to exceptional cases as various studies have highlighted its disadvantages. Seventh and lastly, while saline rinses and topical corticosteroids were widely administered and are recommended, one-third of the participating ENT departments also administered perioperative antibiotics. Further evaluation of this practice is required, as there is currently no evidence for it.

## Data Availability

All data generated or analysed during this study are included in this published article.

## Declarations

The study does not include single patient data, and no involvement of human participants. Participation in the survey is reported anonymously.

## References

[1] Speaker RB, Harney M, Russell J (2023) Choanal Atresia. In: Puri P, Höllwarth M (Eds.) Pediatric Surgery: Diagnosis and Management, Springer Nature Switzerland AG 2009, pp 359–363

[2] Yılmaz Topçuoğlu MS, Schuler PJ, Westhoff JH, Sommerburg O, Wucherpfennig L, Baumann I (2026) Choanal atresia repair in Germany - a comprehensive investigation of the current state of care. Rhinology 64:259–267. 10.4193/Rhin25.318 41486781 10.4193/Rhin25.318

[3] Maas AP, Strieth S, Send T (2023) Klinisches Management der Choanalatresie. Laryngo-Rhino-Otol 103:25–34 10.1055/a-2160-2777 37726020

[4] Moreddu E, Rizzi M, Adil E, Balakrishnan K, Chan K, Cheng A, Daniel SJ, de Alarcon A, Hart C, Hartnick C, Inglis A, Leboulanger N, Pransky S, Rahbar R, Russell J, Rutter M, Sidell D, Smith RJH, Soma M, Spratley J, Thompson D, Trozzi M, Ward R, Wyatt M, Yeung J, Zalzal G, Zur K, Nicollas R (2019) International Pediatric Otolaryngology Group (IPOG) consensus recommendations: Diagnosis, pre-operative, operative and post-operative pediatric choanal atresia care. Int J Pediatr Otorhinolaryngol 123:151–155 31103745 10.1016/j.ijporl.2019.05.010

[5] Urbančič J, Vozel D, Battelino S, Borsos I, Bregant L, Glavan M, Iglic C, Jenko K, Lanisnik B, Kosak S T (2023) Management of Choanal Atresia: National Recommendations with a Comprehensive Literature Review. Child (Basel) 10:91 10.3390/children10010091 PMC9856561 36670642

[6] Yılmaz Topçuoğlu M-S, Hammitsch-Mayer A, Plinkert PK, Baumann I (2024) Versorgung von Choanalatresien in Deutschland. HNO 72:199–203 38189815 10.1007/s00106-023-01410-x PMC10879300

[7] Mir NA, Grewal BS, Kishan J, Elzouki AY, Bhatia JN (1986) Congenital choanal atresia in North African infants. Ann Trop Paediatr 6:141–144 2425723 10.1080/02724936.1986.11748426

[8] Uemura K, Kobayashi M, Otobe Y, Saihara-Nishida E, Takeuchi K (2024) Congenital choanal atresia where the atretic wall thickened while waiting for an elective surgery. Int J Surg Case Rep 119:109737 38714068 10.1016/j.ijscr.2024.109737 PMC11096737

[9] Brihaye P, Delpierre I, De Ville A, Johansson AB, Biarent D, Mansbach AL (2017) Comprehensive management of congenital choanal atresia. Int J Pediatr Otorhinolaryngol 98:9–18 28583512 10.1016/j.ijporl.2017.04.022

[10] Georgiopoulos C, Postler M, Rombaux P, Gudziol V, Abolmaali N, Hummel T (2022) Unilateral Choanal Atresia: Indications of Long-Term Olfactory Deficits and Volumetric Brain Changes Postsurgically. ORL J Otorhinolaryngol Relat Spec 84:89–92 34839294 10.1159/000520188 PMC9153361

[11] Leclerc JE, Leclerc JT, Bernier K (2008) Choanal atresia: long-term follow-up with objective evaluation of nasal airway and olfaction. Otolaryngol Head Neck Surg 138:43–49 18164992 10.1016/j.otohns.2007.09.020

[12] Ferrier S, Hennocq Q, Leboulanger N, Couloigner V, Denoyelle F, Heuzé Y, Khonsari RH (2021) Nasal cavity shape in unilateral choanal atresia and the role of fetal ventilation in facial growth. J Stomatol Oral Maxillofac Surg 122:135–140 32480047 10.1016/j.jormas.2020.05.021

[13] Freng A (1979) Dentofacial development in long-lasting nasal stenosis. Scand J Dent Res 87:260–267 295485 10.1111/j.1600-0722.1979.tb00680.x

[14] Yılmaz Topçuoğlu M-S, Plinkert PK, Federspil PA, Baumann I (2025) Long-term outcome of 56 patients after transnasal endoscopic repair of congenital choanal atresia. Acta Otolaryngol 145:1147–1154 41249090 10.1080/00016489.2025.2561913

[15] Tesoro S, Marchesini L, De Robertis E (2019) Pediatric Anesthesia. Transl Med UniSa 20:1–3 31850244 PMC6910147

[16] Baumann I, Sommerburg O, Amrhein P, Plinkert PK, Koitschev A (2018) Diagnostik und Management der Choanalatresie. HNO 66:329–338 29500502 10.1007/s00106-018-0492-7

[17] Kwong KM (2015) Current Updates on Choanal Atresia. Front Pediatr 3:52 26106591 10.3389/fped.2015.00052 PMC4460812

[18] Brockow I, Söhl K, Hanauer M, Heißenhuber A, Marzi C, Am Zehnhoff-Dinnesen A, Matulat P, Mansmann U, Nennstiel U (2023) [Newborn hearing screening in Germany-results of the 2011/2012 and 2017/2018 evaluations]. Bundesgesundheitsblatt Gesundheitsforschung Gesundheitsschutz 66:1259–1267 37843595 10.1007/s00103-023-03779-0 PMC10622351

[19] Neumann K, Mathmann P, Chadha S, Euler HA, White KR (2022) Newborn hearing screening benefits children, but global disparities persist. J Clin Med 11:271 10.3390/jcm11010271 PMC8746089 35012010

[20] Šebová I, Vyrvová I, Barkociová J (2021) Nasal Cavity CT Imaging Contribution to the Diagnosis and Treatment of Choanal Atresia. Med (Kaunas) 57:93 10.3390/medicina57020093 PMC7909760 33494264

[21] Fitzpatrick NS, Bartley AC, Bekhit E, Berkowitz RG (2018) Skull base anatomy and surgical safety in isolated and CHARGE-associated bilateral choanal atresia. Int J Pediatr Otorhinolaryngol 115:61–64 30368396 10.1016/j.ijporl.2018.09.009

[22] Granata C, Sofia C, Francavilla M, Kardos M, Kasznia-Brown J, Nievelstein RA, Olteanu BS, Owens C, Salerno S, Sorantin E, Apine I (2025) Let’s talk about radiation dose and radiation protection in children. Pediatr Radiol 55:386–396 39095613 10.1007/s00247-024-06009-0

[23] Liktor B, Csokonai LV, Gerlinger I (2001) A new endoscopic surgical method for unilateral choanal atresia. Laryngoscope 111:364–366 11210891 10.1097/00005537-200102000-00033

[24] Bajin MD, Önay Ö, Günaydın RÖ, Ünal OF, Yücel OT, Akyol U, Aydın C (2021) Endonasal choanal atresia repair; evaluating the surgical results of 58 cases. Turk J Pediatr 63:136–140 33686836 10.24953/turkjped.2021.01.016

[25] Ferlito S, Maniaci A, Dragonetti AG, Cocuzza S, Lechien JR, Calvo-Henriquez C, Maza-Solano J, Locatello LG, Caruso S, Nocera F, Achena A, Mevio N, Mantini G, Ormellese G, Placentino A, La Mantia I (2022) Endoscopic Endonasal Repair of Congenital Choanal Atresia: Predictive Factors of Surgical Stability and Healing Outcomes. Int J Environ Res Public Health 19:9084 35897454 10.3390/ijerph19159084 PMC9329715

[26] Maheshwaran S, Pookamala S, Vijay Pradap R, Rajavel S (2023) Practical Tips for Surgical Management of Bilateral Choanal Atresia. Indian J Otolaryngol Head Neck Surg 75:S768–S773 10.1007/s12070-022-03333-5 PMC10188672 37206801

[27] Wormald PJ, Zhao YC, Valdes CJ (2016) The endoscopic transseptal approach for choanal atresia repair. Int Forum Allergy Rhinol 6:654–660 26879228 10.1002/alr.21716

[28] Karligkiotis A, Farneti P, Gallo S, Pusateri A, Zappoli-Thyrion F, Sciarretta V, Pagella F, Castelnuovo P, Pasquini E (2017) An Italian multicentre experience in endoscopic endonasal treatment of congenital choanal atresia: Proposal for a novel classification system of surgical outcomes. J Craniomaxillofac Surg 45:1018–1025 28476356 10.1016/j.jcms.2017.03.015

[29] Yılmaz Topçuoğlu M-S, Schuler PJ, Westhoff JH, Sommerburg O, Baumann I (2025) A recurrence analysis in patients with congenital choanal atresia. J Craniomaxillofac Surg 53:1571–1576 40628561 10.1016/j.jcms.2025.07.003

[30] Alsubaie HM, Almosa WH, Al-Qahtani AS, Margalani O (2021) Choanal Atresia Repair With Stents and Flaps: A Systematic Review Article. Allergy Rhinol 12:1–13 10.1177/21526567211058052 PMC8842470 35173993

[31] Nour YA, Foad H (2008) Swinging door flap technique for endoscopic transeptal repair of bilateral choanal atresia. Eur Arch Otorhinolaryngol 265:1341–1347 18379813 10.1007/s00405-008-0654-4

[32] Rodriguez H, Cuestas G, Passali D (2014) A 20-year experience in microsurgical treatment of choanal atresia. Acta Otorrinolaringol Esp 65:85–92 24556158 10.1016/j.otorri.2013.09.005

[33] Wang P-p, Tang L-x, Zhang J, Yang X-j, Zhang W, Han Y, Xiao X, Ni X, Ge W-t (2021) Combination of the endoscopic septonasal flap technique and bioabsorbable steroid-eluting stents for repair of congenital choanal atresia in neonates and infants: A retrospective study. J Otolaryngol - Head Neck Surg 50:51 34384505 10.1186/s40463-021-00535-9 PMC8361633

[34] Yaniv E, Hadar T, Shvero J, Stern Y, Raveh E (2007) Endoscopic transnasal repair of choanal atresia. Int J Pediatr Otorhinolaryngol 71:457–462 17207539 10.1016/j.ijporl.2006.11.012

[35] Aldriweesh B, Alshareef W, Alsini A, Aljasser A, Alammar A (2022) Safety profile and efficacy of high-dose topical mitomycin-C for choanal atresia repair: A prospective cohort study. Int J Pediatr Otorhinolaryngol 159:111190 35660193 10.1016/j.ijporl.2022.111190

[36] Di Gioia S, Cosseron S, Simon F, Couloigner V, Luscan R (2022) Endoscopic endonasal repair of bilateral choanal atresia in a 1200 g preterm infant: Is it feasible? (With video). Am J Otolaryngol 44:103749 36577169 10.1016/j.amjoto.2022.103749

[37] Cedin AC, Fujita R, Cruz OL (2006) Endoscopic transeptal surgery for choanal atresia with a stentless folded-over-flap technique. Otolaryngol Head Neck Surg 135:693–698 17071296 10.1016/j.otohns.2006.05.009

[38] Saraniti C, Santangelo M, Salvago P (2017) Surgical treatment of choanal atresia with transnasal endoscopic approach with stentless single side-hinged flap technique: 5 year retrospective analysis. Braz J Otorhinolaryngol 83:183–189 27174773 10.1016/j.bjorl.2016.03.009 PMC9442731

[39] Tomoum MO, Askar MH, Mandour MF, Amer MA, Saafan ME (2018) Stentless mirrored L-shaped septonasal flap versus stented flapless technique for endoscopic endonasal repair of bilateral congenital choanal atresia: a prospective randomised controlled study. J Laryngol Otol 132:329–335 29335043 10.1017/S0022215117002614

[40] Strychowsky JE, Kawai K, Moritz E, Rahbar R, Adil EA (2016) To stent or not to stent? A meta-analysis of endonasal congenital bilateral choanal atresia repair. Laryngoscope 126:218–227 26014684 10.1002/lary.25393

[41] Gundle L, Ojha S, Hendry J, Rosen H (2021) Stenting versus stentless repair for bilateral choanal atresia: A systematic review of the literature. Int J Pediatr Otorhinolaryngol 151:110926 34624631 10.1016/j.ijporl.2021.110926

[42] Chowdhury R, Almutawa D, Almutairi NK, Orishchak O, Almhanedi H, Tewfik MA, Daniel SJ (2025) Stented and non-stented endoscopic techniques in bilateral choanal atresia repair: A systematic review and meta-analysis. Int J Pediatr Otorhinolaryngol 196:112494 40701136 10.1016/j.ijporl.2025.112494

[43] Attya H, Callaby M, Thevasagayam R (2021) Choanal atresia surgery: outcomes in 42 patients over 20 years and a review of the literature. Eur Arch Otorhinolaryngol 278:2347–2356 33386964 10.1007/s00405-020-06506-6

[44] Ledderose GJ, Havel M, Ledderose C, Betz CS (2021) Endoscopic endonasal repair of complete bilateral choanal atresia in neonates. Eur J Pediatr 180:2245–2251 33709157 10.1007/s00431-021-04020-3

[45] Saleh AM, Torres KM, Murad MH, Erwin PJ, Driscoll CL (2012) Prophylactic perioperative antibiotic use in endoscopic sinus surgery: a systematic review and meta-analysis. Otolaryngol Head Neck Surg 146:533–538 22241787 10.1177/0194599811434117

